# Activation of the Melanocortin-1 Receptor by NDP-MSH Attenuates Oxidative Stress and Neuronal Apoptosis through PI3K/Akt/Nrf2 Pathway after Intracerebral Hemorrhage in Mice

**DOI:** 10.1155/2020/8864100

**Published:** 2020-11-12

**Authors:** Siming Fu, Xu Luo, Xuan Wu, Tongyu Zhang, Linggui Gu, Yiying Wang, Meng Gao, Yuan Cheng, Zongyi Xie

**Affiliations:** ^1^Department of Neurosurgery, The Second Affiliated Hospital of Chongqing Medical University, Chongqing 400010, China; ^2^Department of Neurosurgery, Xuanwu Hospital of Capital Medical University, Beijing 100053, China; ^3^Department of Human Anatomy, Chongqing Medical University, Chongqing 400016, China

## Abstract

Oxidative stress and neuronal apoptosis play crucial roles in secondary brain injury (SBI) after intracerebral hemorrhage (ICH). Recently, Nle4-D-Phe7-*α*-melanocyte-stimulating hormone (NDP-MSH), a synthetic agonist of the melanocortin-1 receptor (Mc1r), has been proved to inhibit neuroinflammatory in several diseases. This study is aimed at exploring if NDP-MSH could reduce oxidative stress and neuronal apoptosis following ICH, as well as the potential mechanism. A mouse ICH model was induced by autologous blood injection. NDP-MSH was intraperitoneally injected at 1 h after ICH. Mc1r siRNA and PI3K inhibitor LY294002 were administrated to inhibit the expression of Mc1r and phosphorylation of PI3K, respectively. Neurological test, brain water content, enzyme-linked immunosorbent assay (ELISA), terminal deoxynucleotidyl transferase-mediated dUTP-biotin nick end labeling (TUNEL), immunofluorescence, and Western blot analysis were utilized in this study. The results exhibited that Mc1r was mainly expressed in neurons, and its level in the ipsilateral hemisphere was significantly elevated after ICH. NDP-MSH treatment significantly attenuated the neurological deficits and brain water content 24 hours after ICH, which was accompanied by the inhibition of oxidative stress and neuronal apoptosis. The administration of NDP-MSH after ICH significantly promoted the expression of Mc1r, p-PI3K, p-Akt, and p-Nrf2, followed by an increase of Bcl-2 and reduction of cleaved caspase-3. Conversely, downregulating the expression of Mc1r and phosphorylation of PI3K aggravated the neurological deficits and brain edema at 24 hours after ICH, meanwhile, the effect of NDP-MSH on the expression of Mc1r, p-PI3K, p-Akt, p-Nrf2, Bcl-2, and cleaved caspase 3 was also abolished. In conclusion, our data suggest that the activation of Mc1r by NDP-MSH ameliorates oxidative stress and neuronal apoptosis through the PI3K/Akt/Nrf2 signaling pathway after ICH in mice.

## 1. Introduction

Intracerebral hemorrhage remains the leading cause of morbidity and mortality throughout the world, which accounts for 15-20% of all cerebrovascular accidents [1, 2]. Increasing evidence shows that oxidative stress and neuronal apoptosis play pivotal roles in the pathophysiological processes of the secondary brain injury following ICH, and inhibition of oxidative stress and apoptosis can significantly reduce SBI [3–5].

NDP-MSH, an analog of the natural *α*-Melanocyte stimulating hormone, has been proven to exert neuroprotective effects in diverse central nervous system (CNS) diseases, including Alzheimer's disease, intracerebral hemorrhage, and subarachnoid hemorrhage [6–8]. In the CNS, NDP-MSH functions by binding to the G protein-coupled receptors known as melatonin receptors [9–11]. Among them, Mc1r shows specific high-affinity to NDP-MSH and is verified to inhibit cell apoptosis through the PI3K/Akt signaling pathway [12, 13]. To date, the antiapoptotic effects mediated by Mc1r have been demonstrated in the eye, kidney, and tumor tissue [14, 15]. Moreover, it has been determined that Mc1r can reduce neuroinflammation and blood-brain barrier destruction after ICH in mice [7]. However, little about the effect of NDP-MSH on oxidative stress and neuronal apoptosis following ICH is known.

The PI3K signaling pathway regulates diverse cellular functions, including apoptosis, autophagy, cell proliferation, and survival [16]. Previous studies have suggested that the PI3K/Akt pathway is stimulated by the activation of Mc1r in murine B16F10 melanoma cells [17]. In ischemic brain injury, the PI3K/Akt pathway is involved in the neuroprotective effects by activating the Nrf2 signaling pathway [18–20]. And its antiapoptotic effect may be associated with the transcriptional activity of Nrf2.

In this study, we hypothesized that the activation of Mc1r by NDP-MSH could reduce oxidative stress and neuronal apoptosis after experimental ICH, and the potential mechanism might be mediated by the PI3K/Akt/Nrf2 signaling pathway.

## 2. Methods and Materials

### 2.1. The Experimental Animals

A total of 211 C57BL/6 mice (male, 25-30 g) were purchased from the animal center of Chongqing Medical University and were kept in a room with a controlled 12 h light/dark cycle, room temperature, and relative humidity. Mice had free access to food and water. All the protocols in this study were approved by the animal ethics committee of Chongqing Medical University. All procedures were performed following the NIH guidelines for the care and use of laboratory animals published by the National Academy of Sciences.

### 2.2. Experimental Design

**Figure 1:**
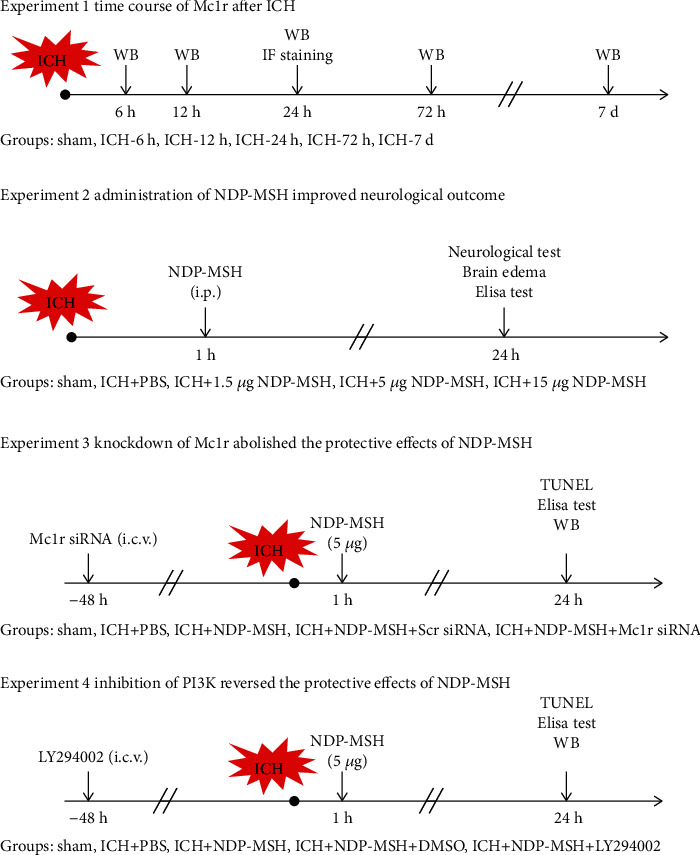
Experimental design and animal groups. ICH: intracerebral hemorrhage; Mc1r: melanocortin-1 receptor; IF staining: immunofluorescence staining; WB: Western blot; Scr siRNA: scrambled siRNA.

Four separate experiments were performed as follows (Figure 1).

1. To explore the temporal expression and cellular localization of Mc1r in perihematoma brain tissue, mice were randomly divided into 6 groups: sham, ICH-6 h, ICH-12 h, ICH-24 h, ICH-72 h, and ICH-7 d (*n* = 6 per group). Western blot analysis was utilized to analyze the change of Mc1r expression. Another eight mice were divided into sham and ICH-24 h (*n* = 4 per group) for double-labeled immunofluorescence staining
2. To evaluate the effect of NDP-MSH on secondary brain injury after ICH, three doses of NDP-MSH (1.5, 5, and 15 *μ*g/mouse, Anaspec, USA) were dissolved in phosphate buffer solution (PBS) and intraperitoneally injected. Mice were randomly divided into the following groups (*n* = 6 per group): sham, ICH + PBS, ICH + NDP-MSH (1.5 *μ*g/mouse), ICH + NDP-MSH (5 *μ*g/mouse), and ICH + NDP-MSH (15 *μ*g/mouse). The neurological tests, brain water content, and oxidative stress were evaluated at 24 hours after ICH. According to the results of neurological tests, the optimal dose of NDP-MSH was determined for the following research
3. To evaluate the effect of activation of Mc1r on SBI after ICH, mice were randomly divided into five groups (*n* = 6): sham, ICH + PBS, ICH + NDP-MSH, ICH + NDP-MSH + Scr siRNA, and ICH + NDP-MSH + Mc1r siRNA. Neurological tests, ELISA, TUNEL, and Western blot were performed at 24 h after ICH
4. To investigate the effect of PI3K on SBI after ICH, mice were divided into the following five groups (*n* = 6): sham, ICH + PBS, ICH + NDP-MSH, ICH + NDP-MSH + saline, and ICH + NDP-MSH + LY294002. PI3K inhibitor LY294002 was injected into the ventricle at 1 h after ICH. Neurological tests, ELISA, and western blot were performed at 24 h after ICH

### 2.3. Induction of ICH Model

The animal ICH model was induced by autologous blood injection as in previous studies [21]. The mice were anesthetized with pentobarbital (40 mg/kg, intraperitoneal injection) and placed in the stereotactic head frame in a prone position. A hole about 1 mm in diameter was drilled at the right side of the bregma (2 mm lateral to the bregma). Then, 30 *μ*L autogenous arterial blood without anticoagulation was collected from the caudal artery and rapidly injected into the basal ganglia (2.3 mm lateral, 0.2 mm anterior to the bregma). Firstly, 5 *μ*L arterial blood was injected at a depth of 2.8 mm from the dura. And five minutes later, the remaining 25 *μ*L of blood was delivered at a depth of 3.5 mm. The needle was kept in the brain for an extra 10 min and withdrawn at a speed of 0.5 mm/min. Finally, the hole was covered with medical bone wax. The animals of the sham group were delivered an equal volume of sterile saline in the same position.

### 2.4. Intracerebroventricular Injection

The intracerebroventricular injection was performed as previously described [22]. The mice were anesthetized with pentobarbital and placed in the stereotactic head frame in a prone position. A longitudinal incision along the central line was made, followed by a burr hole being drilled on the right side of the bregma (1.0 mm lateral to the bregma). According to the manufacturer's instructions, the Mc1r siRNA (100 pmol/2 *μ*l, MSS275666, Thermo Fisher Scientific, USA) or scrambled siRNA was dissolved in nuclease-free water and delivered into the ipsilateral ventricle at a depth of 2.5 mm. Then, the needle was kept in the ventricle for another 5 minutes to prevent possible leakage and slowly pulled out at a speed of 1 mm/min. The burr hole was covered with bone wax, and the incision was stitched. Finally, the mice were placed in separate recovery cages. LY294002 was injected into the ipsilateral ventricle in the same way (Selleck Chemicals, Houston, TX, USA).

### 2.5. Neurological Score

As mentioned above, neurobehavioral functions were blindly assessed at 24 h after ICH by using the modified Garcia test and beam balance test [23]. The modified Garcia test included six subtests: spontaneous movement (0-3), limb symmetry (0-3), forward extension (0-3), climbing (1-3), body proprioception (1-3), and response to tentacles (1-3). In the beam balance test, mice were placed on the beam and were allowed to walk on the beam. The performance of mice was recorded and rated from 0 to 4.

### 2.6. Brain Water Content

Brain water content was assessed 24 h after ICH [24]. The mice were sacrificed under deep anesthesia. The brain was immediately removed and divided into five parts, including bilateral basal ganglia (BG), bilateral cortex (CX), and cerebellum. Each component of the samples was weighed immediately on the electronic analytical balance (FA2204B, Techcomp, USA) to gain the wet weight and then dry at 105°C for 72 h to measure the dry weight. Brain water content (%) was calculated as [(wetweight − dryweight)/wetweight] × 100%.

### 2.7. Histological Analysis

After anesthesia, mice were transcardially perfused with 20 ml ice-cold PBS at 24 h after ICH, followed by perfusion with 20 ml 4% paraformaldehyde. The intact brains were rapidly collected and then fixed with 4% paraformaldehyde at 4°C for 24 h, subsequently immersed in a 20% sucrose solution until they sank to the bottom. Then, the samples were put into the 30% sucrose solution for 24 h. The samples were frozen in a refrigerator at -20°C and sliced into 10 mm thick coronal sections by using a cryostat (CM1860, Leica Microsystems, Germany).

### 2.8. Immunofluorescence Staining

The sections were incubated with 0.3% Triton X-100 for 5 min at room temperature. After being blocked by 5% donkey serum for 1 h, brain sections were incubated with primary antibodies overnight at 4°C. The primary antibodies used for immunofluorescence staining were shown as follows: anti-NeuN (1 : 100, Abcam, ab104224) and anti-Mc1r (1 : 50, Genetex, GTX108190). The sections were then washed with PBS and incubated with appropriate fluorescence-conjugated secondary antibodies (1 : 200, Bioss) at 37°C for 2 h. Microphotographs were visualized and photographed with a fluorescence microscope (U-HGLGPS, OLYMPUS, Japan).

### 2.9. TUNEL Staining

To detect the neuronal apoptosis, TUNEL staining was performed at 24 h after ICH according to the manufacturer's protocol (Roche, Basel, Switzerland). Microphotographs were visualized and photographed with a fluorescence microscope (U-HGLGPS, OLYMPUS, Japan). The number of TUNEL-positive neurons in the perihematomal brain tissue was counted manually. The final data is expressed as the number of TUNEL-positive neurons per square millimeter [25].

### 2.10. ELISA

The perihematomal brain tissue was collected at 24 h following ICH. When the mice were deeply anesthetized, the mice were sacrificed, and the perihematomal brain tissue was removed. After homogenization, the supernatant of samples was collected and centrifuged at 4°C and 3000 revolutions per minute (rpm) for 20 min. According to the manufacturer's instructions, the concentrations of malondialdehyde (MDA), catalase (CAT), and superoxide dismutase (SOD) in the perihematomal brain tissue were measured by ELISA kits (Abcam, USA). The output was measured immediately using an automatic microplate reader at 450 nm [26].

### 2.11. Western Blot Analysis

Mice were perfused with ice-cold PBS at 24 h after ICH. The tissues surrounding the hematoma were collected. The western blot was processed as described previously [27]. After extraction of protein samples, the same amount of protein samples was added into the lanes. Proteins were separated by SDS-PAGE gel electrophoresis and then transferred onto a nitrocellulose membrane. The membrane was incubated with primary antibodies at 4°C overnight or room temperature for 8 hours. The following primary antibodies were used: anti-Mc1r (1 : 500, GTX108190, Genetex), anti-PI3K (1 : 1000, Cell signaling, USA), anti-Akt (1 : 1000, Cell signaling, USA), anti-Nrf2 (1 : 1000, Cell signaling, USA), anti-phospho-PI3K (1 : 1000, Cell signaling, USA), anti-phospho-Akt (1 : 1000, Cell signaling, USA), anti-phospho-Nrf2 (1 : 1000, Cell signaling, USA), anti-Bcl-2 (1 : 1000, Cell signaling, USA), anti-caspase-3 (1 : 1000, Cell signaling, USA), cleaved caspase-3 (1 : 1000, Cell signaling, USA), and anti-*β*-Tubulin (1 : 5000, Santa Cruz, USA). The membrane was washed with washing buffer and then incubated with optimal secondary antibodies (ZSGB-BIO, China) at 37°C for 1 h. The bands were detected with an ECL Plus chemiluminescence reagent kit (Amersham Biosciences, USA). ImageJ software (ImageJ 1.5, NIH, USA) was applied to analyze the relative density of proteins.

### 2.12. Statistical Analysis

All the data were expressed as mean ± standarddeviation (SD) and analyzed using GraphPad Prism 7.0 (GraphPad Software, San Diego, CA, USA). The Shapiro-Wilk normality test was performed to determine the normality of data. For data conforming to the normal distribution, a one-way analysis of variance (one-way ANOVA) was used to evaluate the statistical difference followed by a Tukey multiple comparison post hoc analysis. For data that does not conform to the normal distribution, Kruskal-Wallis one-way ANOVA on Ranks was performed, followed by Tukey's multiple comparison post hoc analysis. The statistical differences between the two groups were analyzed using Student's unpaired or two-tailed *t*-test. *p* < 0.05 was defined as statistically significant.

## 3. Results

### 3.1. Mortality and Exclusion

In total, 211 male mice were used in this study. The overall mortality was 9.48% (20/211). None of the mice in the sham group died. Seven mice were excluded from this study because of no hematoma in basal ganglia (Table 1).

### 3.2. Expression of Mc1r in the Perihematomal Brain Tissue at 24 h after ICH

**Figure 2:**
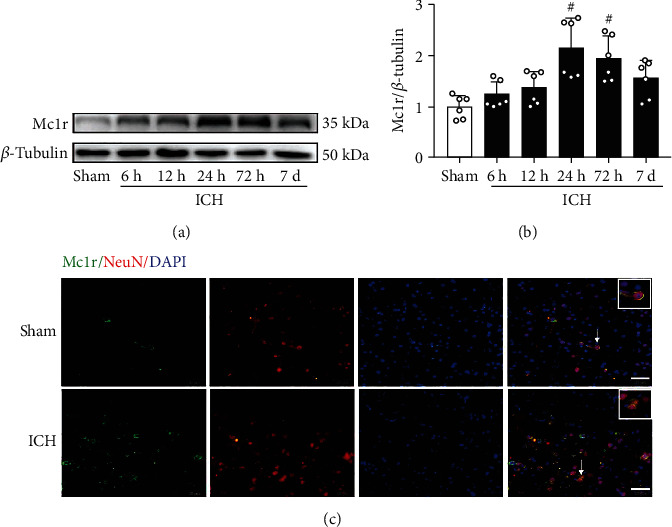
Endogenous expression of Mc1r after ICH. (a, b) Representative Western blot bands and quantitative analysis of temporal expression of Mc1r in the perihematomal brain tissue after ICH. #*p* < 0.05 vs. sham group. *n* = 6 per group. (c) Representative images of immunofluorescence staining showed that Mc1r was colocalized with neurons (NeuN) at 24 h after ICH (white arrow). *n* = 4 per group. Scalebar = 50*μ*m.

The temporal expression of endogenous Mc1r in the perihematomal tissue was evaluated by Western blots. The level of Mc1r was dramatically increased after ICH and reached the highest at 24 h when compared with the sham group (Figures 2(a) and 2(b)). Immunofluorescent staining exhibited that Mc1r (green) was expressed in neurons (red), and, when compared to the sham group, the Mc1r-positive neurons were significantly increased after ICH (Figure 2(c)).

### 3.3. NDP-MSH Significantly Reduced the Neurological Deficits, Brain Edema, and Reactive Oxygen Species (ROS) after ICH

**Figure 3:**
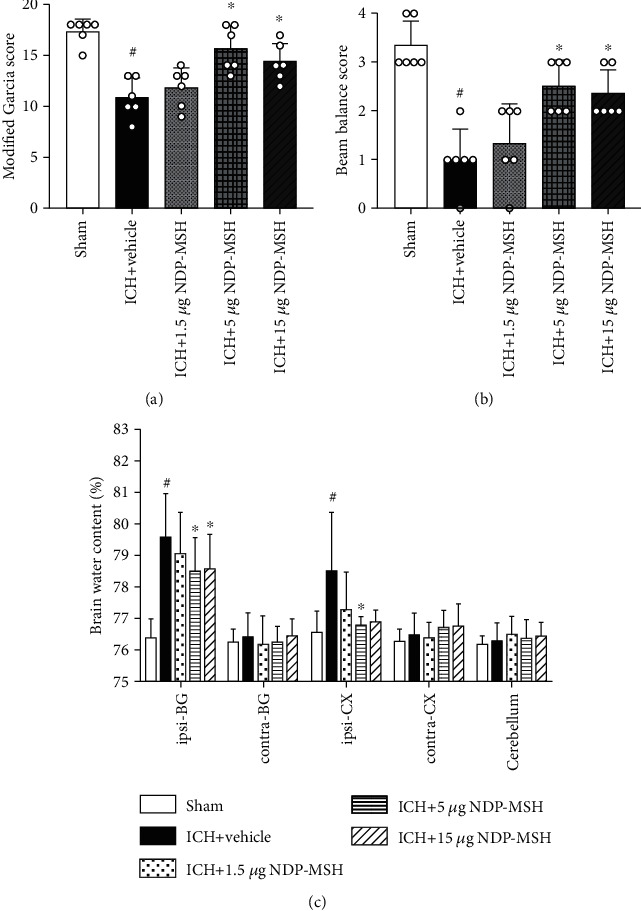
The administration of NDP-MSH improved neurological deficits and brain edema at 24 h after ICH. (a, b) Modified Garcia and beam balance scores showed intraperitoneal administration of NDP-MSH at the dose of 5 or 15 *μ*g/mouse significantly reduced neurological deficits at 24 h after ICH. (c) NDP-MSH treatment attenuated the brain water content at 24 h after ICH. *n* = 6 each group. Ipsilateral basal ganglia (ipsi-BG), contralateral basal ganglia (contra-BG), ipsilateral cortex (ipsi-CX), contralateral cortex (contra-CX), and cerebellum. ^#^*p* < 0.05 vs. sham group; ^∗^*p* < 0.05 vs. ICH + vehicle group.

Compared to the sham group, the scores of modified Garcia and beam balance of the ICH + vehicle group were significantly reduced at 24 h post-ICH (Figures 3(a) and 3(b)). Meanwhile, when compared to the sham group, obvious brain edema was detected in the ipsilateral basal ganglia and cortex of mice from ICH + vehicle group at 24 h after ICH induction (Figure 3(c)). However, the administration of NDP-MSH at the dose of 5 and 15 *μ*g/mouse significantly ameliorated the neurological deficits and brain edema (Figures 3(a)–3(c)). Based on the results, the NDP-MSH dose of 5 *μ*g/mouse was used for the subsequent study.

**Figure 4:**
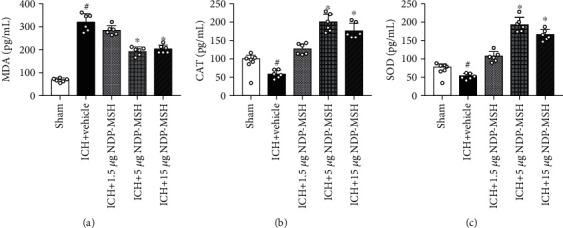
Oxidative stress-related proteins were detected by ELISA at 24 h after ICH. (a–c) Administration of NDP-MSH at the dose of 5 and 15 *μ*g/mouse markedly reduced the MDA level and increased CAT and SOD levels in the perihematomal brain tissue at 24 h after ICH. *n* = 6 per group. ^#^*p* < 0.05 vs. sham group; ^∗^*p* < 0.05 vs. ICH + vehicle. MDA: malondialdehyde; CAT: catalase; SOD: superoxide dismutase.

After ICH, the MDA level in the perihematomal brain tissue was markedly elevated, while the levels of CAT and SOD were significantly reduced when compared to the sham group (Figures 4(a)–4(c)). These detrimental changes were inhibited by the administration of NDP-MSH at the dose of 5 and 15 *μ*g/mouse (Figures 4(a)–4(c)). However, the NDP-MSH delivered at an amount of 1.5 *μ*g/mouse showed no significant difference in the levels of MDA, CAT, and SOD.

### 3.4. NDP-MSH Treatment Inhibited Neuronal Apoptosis after ICH

**Figure 5:**
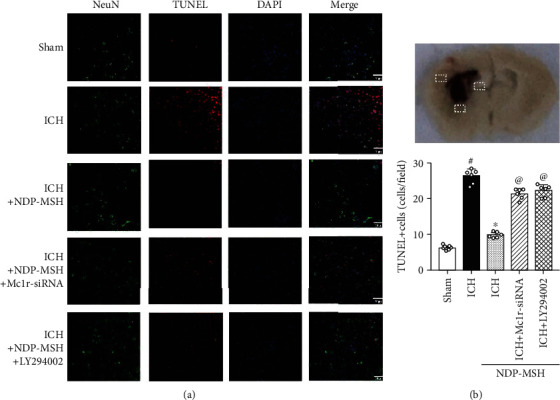
Effects of NDP-MSH on neuronal apoptosis at 24 h after ICH. (a) Representative microphotographs of TUNEL staining at 24 h after ICH. (b) Quantitative analysis of the number of TUNEL-positive neurons. ^#^*p* < 0.05 vs. sham group; ^∗^*p* < 0.05 vs. ICH + vehicle; ^@^*p* < 0.05 vs. ICH + NDP-MSH. *n* = 4 per group, scalebar = 50*μ*m.

TUNEL staining showed that the numbers of TUNEL-positive neurons in the perihematomal brain tissue in the ICH + vehicle group were significantly elevated at 24 h post-ICH when compared to the sham group (Figures 5(a) and 5(b)). And NDP-MSH treatment significantly reduced the numbers of TUNEL-positive neurons in the perihematomal brain tissue at 24 h after ICH (Figures 5(a) and 5(b)). However, knockdown of Mc1r with specific siRNA or inhibiting the PI3K with LY294002 aggravated the neuronal apoptosis (Figures 5(a) and 5(b)).

### 3.5. Knockdown of Endogenous Mc1r Expression Abolished the Effects of NDP-MSH on Oxidative Stress and Neuronal Apoptosis following ICH

**Figure 6:**
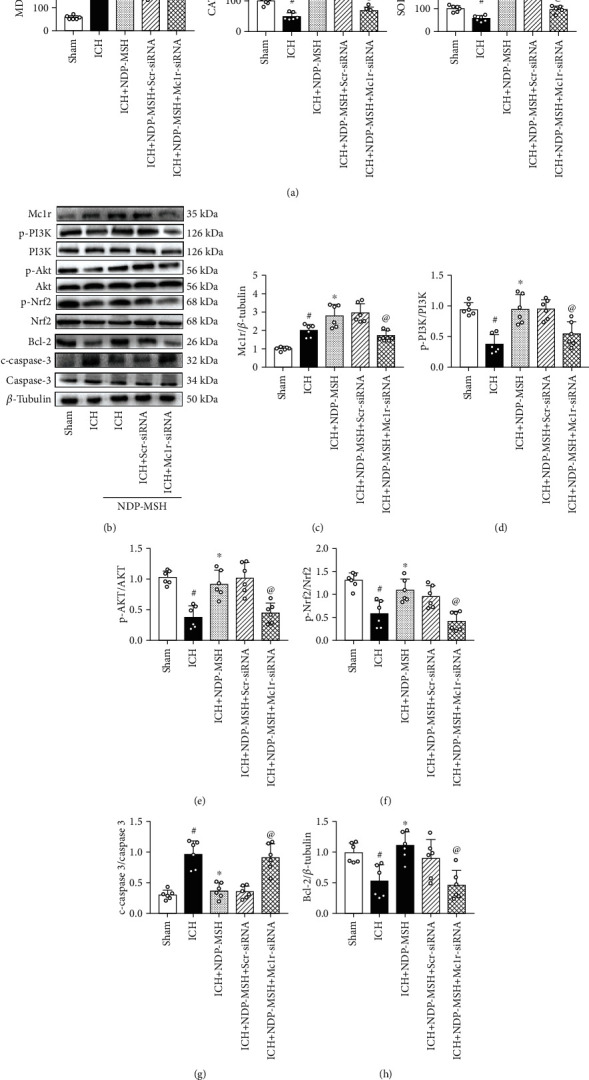
Knockdown of Mc1r expression abolished the effects of NDP-MSH on oxidative stress and neuronal apoptosis following ICH. (a) The changes in the expression levels of MDA, CAT, and SOD in the perihematomal brain tissue after the administration of Mc1r siRNA. Representative Western blot images (b) and quantitative analyses of Mc1r (c), p-PI3K/PI3K (d), p-AKT/AKT (e), p-Nrf2/Nrf2 (f), Bcl-2 (g), and cleaved caspase 3 (h). *n* = 6 per group. ^#^*p* < 0.05 vs. sham; ^∗^*p* < 0.05 vs. ICH; ^@^*p* < 0.05 vs. ICH + NDP-MSH + Scr siRNA. Scr siRNA: scrambled siRNA.

The data of ELISA suggested that when compared to the ICH + NDP-MSH + Scr siRNA group, knockdown of Mc1r results in a significant increase of MDA level, accompanied by a decrease in the CAT and SOD at 24 h post-ICH (Figure 6(a)). Western blot analysis showed, compared to the ICH + vehicle group, Mc1r expression was significantly improved by the NDP-MSH after ICH, which could be inhibited by Mc1r siRNA (Figures 6(b) and 6(c)). Moreover, NDP-MSH treatment also promoted the expression of p-PI3K, p-Akt, p-Nrf2, and Bcl-2 in the perihematomal brain tissue at 24 h post-ICH, accompanied by a decrease of the cleaved caspase 3 (Figures 6(d)–6(h)). Conversely, knockdown of Mc1r using specific siRNA abolished the effect of NDP-MSH on the downstream signaling molecules when compared to the ICH + NDP-MSH + Scr siRNA group (Figures 6(b)–6(h)).

### 3.6. PI3K-Specific Inhibitor LY294002 Reversed the Neuroprotective Effects of NDP-MSH after ICH

**Figure 7:**
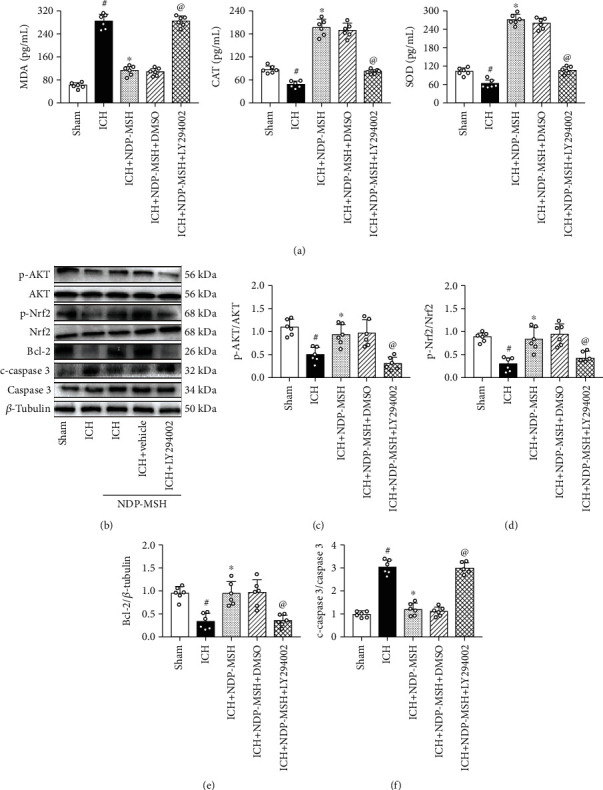
The administration of LY294002 abolished the beneficial effects of NDP-MSH on oxidative stress and neuronal apoptosis. (a) The changes in the expression levels of MDA, CAT, and SOD in the perihematomal brain tissue after the administration of LY294002. Representative Western blot images (b) and quantitative analyses of p-AKT/AKT (c), p-Nrf2/Nrf2 (d), Bcl-2 (e), and cleaved caspase 3 (f). *n* = 6 per group. ^#^*p* < 0.05 vs. sham; ^∗^*p* < 0.05 vs. ICH; ^@^*p* < 0.05 vs. ICH + NDP-MSH + DMSO. LY294002: PI3K inhibitor.

PI3K-specific inhibitor LY294002 was administered by intracerebroventricular injection at 48 h before ICH induction. Compared with the ICH + NDP-MSH + DMSO group, inhibition of PI3K significantly increased the expression of MDA and reduced the levels of SOD and CAT (Figure 7(a)). Furthermore, inhibition of PI3K significantly decreased the expression of p-Akt, p-Nrf2, and Bcl-2 accompanied by an increase of cleaved caspase 3 in the perihematomal brain tissue (Figures 7(b)–7(f)).

## 4. Discussion

In the present study, we verified the neuroprotective effects of NDP-MSH and investigated the underlying mechanism in ICH. Firstly, our research found that the Mc1r was mainly expressed in neurons, and its expression level was significantly increased in the perihematomal brain tissue at 24 h after ICH. Secondly, the intraperitoneal administration of NDP-MSH at a dose of 5 *μ*g/mouse significantly improved the neurological deficits and alleviated the oxidative stress and neuronal apoptosis following ICH. Thirdly, NDP-MSH treatment was associated with the upregulation of p-PI3K, p-Akt, p-Nrf2, and Bcl-2 and downregulation of cleaved-caspase 3 in the perihematomal brain tissue at 24 h after ICH. Finally, we observed that silencing Mc1r by specific siRNA or inhibiting PI3K with LY294002 abolished the beneficial effects of NDP-MSH on neurological functions and expressions of the proteins related to neuronal apoptosis and oxidative stress.

Oxidative stress and neuronal apoptosis are the main pathological changes after ICH [3, 5]. Oxidative stress has been identified as a response to various harmful stimuli and produces excessive amounts of ROS, which reflects an imbalance between the ROS and the biological ability to scavenge ROS [28, 29]. After ICH, the hemoglobin/haem is released from the lysed red blood cells. It is then engulfed by infiltrated macrophages or microglia in the perihematomal brain region and produced a large amount of ferrous/ferric iron, which results in the formation of ROS and lipid peroxidation [30–33]. Moreover, the excess iron can be transported into neurons and form highly toxic hydroxyl radicals (•OH), which cause damage to the DNA, proteins, and lipid membranes [4, 5]. The free radical cascade, together with neuroinflammation, cytokine stimulation, thrombin, and blood components, contributes to the neuronal apoptosis induced by ICH and regulates the expression of apoptosis-related genes, such as Bcl-x and caspase 3 [3]. Therefore, inhibiting oxidative stress and neuronal apoptosis is a feasible method to reduce secondary brain injury after ICH. Previous studies showed that MDA is a robust marker of lipid peroxidation, and the CAT and SOD are vital antioxidant enzymes that are involved in the metabolism of oxygen-free radicals and prevent the formation of other ROS [34]. Generally, the ROS level can be detected by ELISA assay, staining of 2, 7-dichlorofluorescein diacetate, and nitroblue tetrazolium assay. In this study, the oxidative stress was determined by the level of the MDA, CAT, and SOD in the perihematomal brain tissue, which was measured by the corresponding ELISA kits.

NDP-MSH has been demonstrated to regulate diverse pathophysiological processes in the CNS, including excitatory toxicity, oxidative stress, neuroinflammation, and apoptosis [35]. Following traumatic brain injury, NDP-MSH prevents apoptosis by activating the extracellular signal-regulated kinase 1 and 2 (ERK1/2) and subsequently mediates the caspase-3 cleavage and Bax/Bcl-2 expression [36]. The previous study has shown the antiapoptotic effect of NDP-MSH in the CNS by binding to different melanocortin receptors (Mc1r to Mc5r) [37, 38]. Among the melatonin receptors, Mc1r shows a higher affinity to NDP-MSH than other receptors. And it is widely expressed in neurons of periaqueductal gray, astrocytes, and microvascular endothelial cells [12, 39, 40]. In the present study, we confirmed that Mc1r was mainly expressed in the neurons of mice and significantly increased after ICH. And we demonstrated that the intraperitoneal administration of NDP-MSH could further augment the endogenous expression of Mc1r and inhibit oxidative stress and neuronal apoptosis. Therefore, we hypothesized that the neuroprotective effects of NDP-MSH on the secondary brain injury after ICH might be mediated, in part, in an Mc1r dependent manner.

Nrf2 is the crucial molecules to maintain redox homeostasis under oxidative stress conditions [41]. Under physiological conditions, Nrf2 is constitutively ubiquitinated by Kelch-like ECH-associated protein 1 (Keap1) and degraded by the 26S proteasomal pathway, thus is prevented from entering the nucleus and activating the transcriptional activities [42]. Conversely, under oxidative stress conditions, the dissociation with Keap1 can inhibit the degradation of Nrf2, which allows Nrf2 to enter the nucleus and trigger the expression of a series of genes encoding multiple protective phase II defense enzymes to restore redox homeostasis and thus enhance cell survival [42, 43]. Mitochondria are known as one of the primary sources of cellular ROS production. Nrf2 signaling is observed to counteract the mitochondria-derived ROS [44]. Its mechanism is summarized as the following three aspects. Firstly, Nrf2 activation contributes to elevate the biosynthesis of mitochondrial antioxidants such as glutathione and NADPH (a reducing molecule in cells), which mediates the removal of hydrogen peroxide [45, 46]. Secondly, Nrf2 directly induces the expression of some mitochondrial antioxidant enzymes, such as superoxide dismutase 2 (SOD2), peroxiredoxin 3 (Prdx3), Prdx5, GPx1, and thioredoxin reductase 2 (TrxR2) [47]. Finally, Nrf2 activation upregulates the levels of nuclear respiratory factor-1 (Nrf1) and the mitochondrial transcription factor A (TFAM), which is associated with the expression of mitochondrial respiratory subunits and translational components [47].

The PI3K pathway regulates the Nrf2-dependent inducible expression of the antioxidative proteins, including heme oxygenase-1 (HO-1), thioredoxin, and peroxiredoxin-I [41, 48]. A study in experimental ischemic stroke also has observed that the activation of Nrf2 inhibits the oxidative stress injury by promoting the expression of HO-1 [18]. Our present data showed that following the administration of NDP-MSH, PI3K/Akt signaling pathway was activated by Mc1r and protected neurons against ICH-induced oxidative stress and apoptosis by promoting p-Nrf2 and Bcl-2 and inhibiting cleaved caspase 3 expression. On the contrary, when PI3K/Akt signaling was blocked by PI3K inhibitor LY294002, the antioxidant and antiapoptotic effects of NDP-MSH were abolished, due to the reverse of the changes in p-Nrf2, Bcl-2, and caspase 3 expression. Thus, our results implied that the neuroprotective effect of NDP-MSH after ICH was mediated by Mc1r and through the PI3K/Akt/Nrf2 signaling pathway.

Some limitations existed in this study. Firstly, although our results have verified that the neuroprotective effect of NDP-MSH was mediated by Mc1r, NDP-MSH can also bind to Mc3r and Mc4r, whether it exerts some neuroprotective effects remains to be further explored. Secondly, the neuroprotective role of NDP-MSH in CNS diseases was regulated through multiple pathways (CREB, MAPK, etc.) [6, 48], which can influence the neurological outcome after ICH as well. Finally, we only studied the neuroprotective effect of NDP-MSH on oxidative stress and neuronal apoptosis induced by ICH, but other pathological processes might be involved, such as autophagy, neuroinflammation, and pyroptosis.

## 5. Conclusion

NDP-MSH alleviates oxidative stress and neuronal apoptosis and improves neurological impairments after ICH in mice. The neuroprotective role of NDP-SMH is mediated, at least in part, via the PI3K/Akt/Nrf2 signaling pathway. Therefore, NDP-MSH might be a potential therapeutic agent for ICH patients.

## Figures and Tables

**Table 1:** Experimental groups and mortality rate.

Experimental groups	Neurological test Brain water content	ELISA	IF staining	WB	TUNEL	Exclusion	Mortality	Subtotal
Experiment 1								
Sham			4	6		0	0	10
ICH (6 h, 12 h, 24 h, 72 h, and 7 d)			4	6 × 5		3	6 (13.95%)	43
Experiment 2								
Sham	6	6				0	0	12
ICH + PBS	6	6				0	2 (14.29%)	14
ICH + NDP-MSH (1.5 *μ*g/mouse)	6	6				0	2 (14.29%)	14
ICH + NDP-MSH (5 *μ*g/mouse)	6	6				0	0	12
ICH + NDP-MSH (15 *μ*g/mouse)	6	6				2	2 (12.5%)	16
Experiment 3								
Sham					4	0	0	4
ICH + PBS				6	4	0	0	10
ICH + NDP-MSH				6	4	0	1 (9.09%)	11
ICH + NDP-MSH + Scr siRNA		6		6		0	0	12
ICH + NDP-MSH + Mc1r siRNA		6		6	4	1	4 (19.05%)	21
Experiment 4								
ICH + NDP-MSH + DMSO		6		6		0	1 (7.69%)	13
ICH + NDP-MSH + LY294002		6		6	4	1	2 (10.53%)	19
Total	30	54	8	72	20	7	20 (9.48%)	211

ICH: intracerebral hemorrhage; PBS: phosphate-buffered saline; Mc1r: melanocortin-1 receptor; LY294002: PI3K specific inhibitor; siRNA: small interfering RNA.

## Data Availability

The data used to support the findings of this study are available from the corresponding author.
